# Staged repair of pulmonary atresia, ventricular septal defect, and major systemic to pulmonary artery collaterals

**Published:** 2010

**Authors:** Sachin Talwar, Rachit Saxena, Shiv Kumar Choudhary, Balram Airan

Ventricular septal defect (VSD) with pulmonary atresia (PA) can be considered to be the severest form of tetrology of Fallot wherein the right ventricular outflow tract obstruction has progressed to the extent of atresia. This atresia can occur either at the infundibulum or as a plate atresia of the pulmonary valve. An important observation is that the plate-type atresia is more frequently associated with well-developed pulmonary arteries. The other significant abnormality in patients with VSD and pulmonary atresia (PA) is the presence of arborization abnormalities. The blood supply to a particular lung segment can be derived from a systemic artery or a central pulmonary artery or a combination of both. These major aorto pulmonary collaterals (MAPCAS) expose a particular lung segment to the systemic arterial pressure and produce local pulmonary vascular changes akin to pulmonary arterial hypertension or, paradoxically, the MAPCA may develop significant proximal stenosis over a period of time, and thereby, prevent the development of local pulmonary vascular changes. It is important to identify the extent of structural abnormality of the central pulmonary arteries, as these are crucial in planning the management strategy. These may be confluent, nonconfluent or totally absent. When present their size may be highly variable and hence of surgical significance.

## SURGICAL APPROACHES

VSD with PA was first corrected by Lillehei[1] in 1955 using controlled cross-circulation and a series of 10 cases was reported. Since then, the management strategy has evolved considerably. Initially the popular approach was “staged” in which following an initial surgical palliation to relieve hypoxia, a unifocalization procedure was done. This was followed by the placement of a right ventricle (RV) to pulmonary artery conduit and closure of the VSD. More recently, single stage unifocalization, VSD closure, and RV to pulmonary artery conduit placement has become a popular approach with many groups as illustrated in the accompanying paper by Murthy *et al*.[2] In those who can not be successfully palliated by either of these approaches, heart lung transplant remains the only option. However, it is extremely difficult to make generalizations and the initial procedure of choice varies from patient to patient depending upon the anatomy of central pulmonary artery, arborization abnormality, age of the patient etc. The accompanying paper has already discussed the single-stage approach in great detail, so, it may be pertinent here to discuss the staged approach.

The aims of the staged approach in a patient with VSD, PA and MAPCAs are (a) to increase the central pulmonary artery flow by establishing a direct continuity between the ascending aorta or the RV and the small pulmonary artery thereby stimulating its growth: Stage I (b) unifocalization of MAPCAS in both lungs: Stage II, and (c) closure of the VSD and establishment of RV to pulmonary artery continuity: Stage III. The principle behind staged procedure is that even very small native central pulmonary arteries, have a potential to grow. Therefore, augmentation of blood flow to the central pulmonary arteries may lead to gradual and better “rehabilitation.” The main advantage of the staged approach is that it breaks the entire procedure into less stressful and better-tolerated smaller surgical segments. Additionally, management of MAPCAS may be much easier by via a posterolateral thoracotomy than through a sternotomy approach. The palliative procedures have the potential to increase the pulmonary blood flow and promote growth of even diffusely very small right and left pulmonary arteries, which may otherwise not be amenable to direct surgical enlargement.[3] The systemic to pulmonary artery shunt (most often a Blalock–Taussig shunt) enlarges the ipsilateral pulmonary artery to the same extent as the contralateral pulmonary artery.[4] In the presence of short segment PA, an option is to place a transannular patch across the right ventricular outflow tract and in-to the main pulmonary artery. This involves incising the main pulmonary artery longitudinally and extending the incision across the pulmonary annulus into the right ventricular outflow tract. The right ventricular to pulmonary artery continuity is then established by autologous pericardium or low porosity Dacron.[5] Although this procedure is beneficial clinically as evidenced by improved arterial oxygen saturation and decreased hemoglobin values, it has a high incidence of producing stenosis either in the right or the left or both the pulmonary arteries.[6]

**Figure 1:**
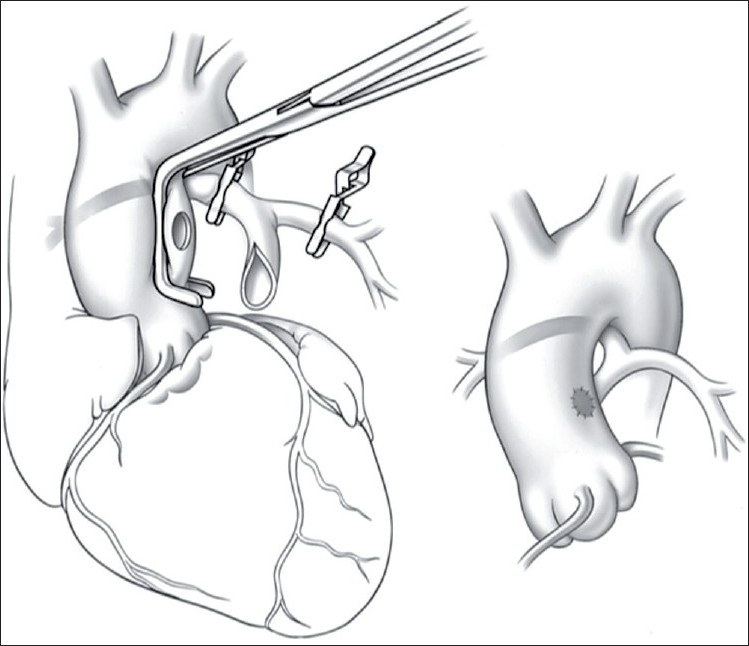
Melbourne shunt: side-biting clamp controls the ascending aorta; soft clamps control the branch pulmonary arteries. *Inset* demonstrates the completed shunt with the pulmonary artery anastomosed to the posterior and left lateral aspect of the ascending aorta close to the sinotubular junction. Reproduced with permission from Duncan *et al*.^7^

Another option is central ascending aorta to main pulmonary artery shunt,[7] (Melbourne shunt Figure 1), which is considered in the presence of confluent hypoplastic pulmonary arteries. In the presence of a tapering main pulmonary artery, it can be detached from the right ventricular outflow tract and anastamosed to the ascending aorta. The central shunts promote a more uniform growth of the pulmonary arteries. However, they may lead to early development of congestive heart failure and pulmonary arterial hypertension and hence the patients need to be followed closely.

The second stage involves MAPCA ligation and transplantation. Ligation is done when there is dual blood supply to the same segment of lung from native pulmonary artery as well as from the MAPCA. MAPCAs need transplantation when they are the only source of blood supply to a broncopulmonary segment and there is no peripheral stenosis and they are not hypertensive.[5]

The MAPCAS can be directly anastamosed end to side with branch pulmonary artery or one MAPCA is anastamosed to the branch pulmonary artery and the others are anastamosed to this MAPCA. Azygos vein has also been used as an interposition graft to transplant MAPCAS.[8]

This approach of staged unifocalization allows the surgeon to tailor the surgical procedure to the specific needs of every patient depending on the existing anatomy and physiology.[7] For patients who have congestive heart failure, unifocalization is initially performed on the side with the least obstructed pulmonary blood flow, which makes congestive heart failure easier to manage and decreases the likelihood of development of obstructive disease in overcirculated pulmonary segments. Patients with significant cyanosis have unifocalization performed initially on the side with the most obstructed MAPCAs. Modified Blalock–Taussig shunt is often performed adjunctively at the time of unifocalization in these cases to further augment pulmonary blood flow with a resulting decrease in cyanosis. Staged unifocalization may not always require bilateral thoracotomies in addition to median sternotomy. Large central MAPCAs that originate relatively close to the central pulmonary arteries (especially those on the left side) may be easily unifocalized at the time of complete repair through a median sternotomy. In the majority of cases all unifocalization and ultimate complete repair can be performed within a year after entering the operative sequence that commences with the performance of a central shunt.

The last stage involves closure of ventricular septal defect and establishment of continuity between the right ventricular outflow tract and pulmonary artery. The Birmingham formula is used preoperatively to calculate the postoperative ratio of the right ventricular to left ventricular pressure (pRV/LV) ratio and patients with pRV/LV ratio < 0.7 are considered suitable for repair.[5]

In a landmark paper on the staged approach,[5] Drs Iyer and Mee reported the algorithm and results of their approach in a series of 58 consecutive patients over a 10-year period. A total of 121 staging procedures were performed with an overall mortality of 10.3%. One hundred thirty-four major collaterals were either ligated or transplanted. Thirty patients eventually underwent complete repair with an early mortality of 3.3% and late mortality of 10.0%. Twenty-six current survivors of repair remained clinically well after a mean follow-up of 3.6 years. Ten patients were in various stages of preparation. Twelve patients (20.7%) failed to achieve minimum requirements for repair after staging and were awaiting further palliation or heart–lung transplantation when this study was published.

**Figure 2:**
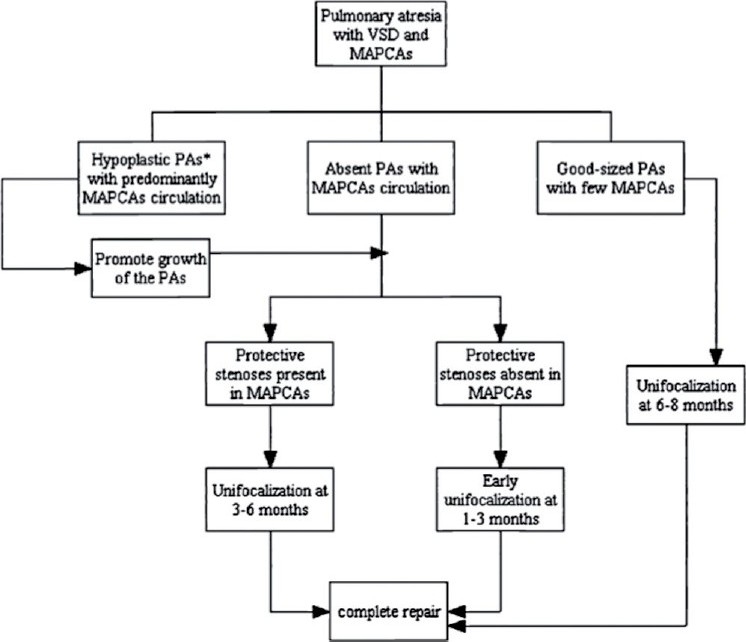
Management algorithm for patients with pulmonary atresia with ventricular septal defect (VSD) and major aortopulmonary collateral arteries (MAPCAs) based on the nature of pulmonary vascular supply. Reproduced with permission from Gupta *et al*.^9^

In a large series from Los Angles[9] involving 104 patients undergoing staged repair, 58 patients (55.765) achieved complete anatomic repair. The mortality in stage 1 repair was 6%, 9% in stage 2 and 8.5% in stage 3. The 10-year mortality was 16.5%. The median pRV/LV was 0.5. The number of collateral vessels incorporated in the repair was an independent risk factor for postoperative mortality and an elevated pRV/LV. The authors had a simple management algorithm for these patients, which is presented in Figure 2.

Recently, the group from Shanghai, China has reported a different two-stage approach.[10] In the first stage, a left thoracotomy is performed along with unifocalization of the left-sided MAPCAs into a vascular graft. A systemic to this vascular graft shunt is added and the other end of the graft is closed to form a caecum, which is then placed under the aortic arch so that it can be approached at the next stage through the midline. At the next stage, 6 to 11 months later, a median sternotomy is performed and the unifocalization of the right sided MAPCAs is achieved into another vascular graft. The previous left shunt is taken down and after establishing cardiopulmonary bypass, the two vascular grafts: right and left are connected to each other and a RV to this graft confluence conduit is placed along with VSD closure. Using this approach, the authors did not have any mortality at the first stage and there was only one death in the 11 patients undergoing completion of the repair at the second stage. There were no late deaths and no reintervention during the mean follow up period of 25.4±15.2 months.

**Figure 3:**
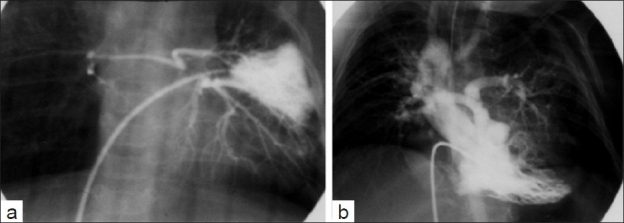
(a) Extremely diminutive central pulmonary arteries (Nakata: 15) showing the ‘‘sea-gull’’ aspect, filled by a retrograde angiogram in a pulmonary vein. A MAPCA is also opacified retrogradely. (b) Three months after RV–PA connection by patch done at 4 months of age, there is a nice development of the PAs, with normal pressures and satisfactory distribution. Reproduced with permission from Metras *et al*.^11^

In another report from France, Metras *et al*. adopted a different staged procedure in patients with extreme hypoplasia of the pulmonary arteries.[11] The initial stage (performed as early as 10 days, range 0.1–18 months) involved rehabilitation of pulmonary arteries by direct continuity between ascending aorta or right ventricle and the diminutive pulmonary arteries, followed by interventional catheterization (stenosis dilatation, pulmonary artery stents and coil occlusion of MAPCAS) and a subsequent complete correction with closure of ventricular septal defect and right ventricle to pulmonary artery conduit. There was 90% survival after the first stage. Seventy percent patients had complete correction. During the follow up of 83±65 months, all patients had improved, 50% had no cardiac medications, none had residual shunt, RV/LV pressure ratio was 0.6 (range 0.3–1). In this series in all cases, the main pulmonary artery branch size was between 1 and 2.7 mm (mean 1.45 mm) and the Nakata index was 3.5–58, mean 20.6 mm^2^/m^2^indicating extreme hypoplasia of the pulmonary arteries. At the second stage, there was satisfactory growth of these pulmonary arteries [Figure 3].

Having summarized the various approaches to the management of VSD, PA, and MAPCAs, one should not forget that there is extreme variation in the anatomy, which will require individualization of the approach to any given patient. At the same time as detailed by Murthy *et al*., in the accompanying paper the single stage approach still has the advantages of preventing development of stenosis in MAPCAS and any pulmonary hypertensive changes, and because it is performed early in life, both MAPCA stenosis and pulmonary arterial hypertension are unlikely to develop by this age. The other advantage of single staged repair is early normalization of physiology and correction of polycythemia and cyanosis during infancy.[12,13] It also avoids long-term cardiac dysfunction due to prolonged cyanosis and arrythmias. However, the superiority of the single over the multiple stage approach will require long-term, prospective, randomized, multicentric trial, which seems to be distant as of now.
